# Enhancement of Energy Expenditure following a Single Oral Dose of Flavan-3-Ols Associated with an Increase in Catecholamine Secretion

**DOI:** 10.1371/journal.pone.0112180

**Published:** 2014-11-06

**Authors:** Yusuke Matsumura, Yuta Nakagawa, Katsuyuki Mikome, Hiroki Yamamoto, Naomi Osakabe

**Affiliations:** Department of Bio-science and Engineering, Shibaura Institute of Technology, Saitama, Saitama, Japan; Sanford-Burnham Medical Research Institute, United States of America

## Abstract

Numerous clinical studies have reported that ingestion of chocolate reduces the risk of metabolic syndrome. However, the mechanisms by which this occurs remain unclear. In this murine study, the metabolic-enhancing activity of a 10 mg/kg mixture of flavan-3-ol fraction derived from cocoa (FL) was compared with the same single dose of (-)-epicatechin (EC). Resting energy expenditure (REE) was significantly increased in mice treated with the FL versus the group administered the distilled water vehicle (Cont) during periods of ad libitum feeding and fasting. Mice were euthanized under the effect of anesthesia 2, 5, and 20 hr after treatment with FL or Cont while subsequently fasting. The mRNA levels of the uncoupling protein-1 (UCP-1) and peroxisome proliferator-activated receptor gamma coactivator-1 alpha (PGC-1α) in brown adipose tissue (BAT) were significantly increased 2 hr after administration of FL. UCP-3 and PGC-1α in the gastrocnemius were significantly increased 2 and 5 hr after administration of the FL. The concentrations of phosphorylated AMP-activated protein kinase (AMPK) 1α were found to be significant in the gastrocnemius of mice 2 and 5 hr after ingesting FL. However, these changes were not observed following treatment with EC. Plasma was collected for measurement of catecholamine levels in other animals euthanized by decapitation 2 and 4 hr after their respective group treatment. Plasma adrenaline level was significantly elevated 2 hr after treatment with FL; however, this change was not observed following the administration of EC alone. The present results indicated that FL significantly enhanced systemic energy expenditure, as evidenced by an accompanying increase in the type of gene expression responsible for thermogenesis and lipolysis, whereas EC exhibited this less robustly or effectively. It was suggested the possible interaction between thermogenic and lipolytic effects and the increase in plasma catecholamine concentrations after administration of a single oral dose of FL.

## Introduction

Flavan-3-ols, a group of polyphenolic substances, are distributed in a number of plant foods such as cocoa beans, red wine, and apples. Of these foods, chocolate is known to be rich in flavan-3-ols, including the flavan-3-ol monomers, (+)-catechin and (−)-epicatechin, and the oligomers, as B-type procyanidins that are linked by C4–C8 bonds [1]–[3]. Recent meta-analyses have suggested that the ingestion of chocolate reduces the risk of cardiovascular diseases [4], [5]. These reports have shown that chocolate consumption was associated with a considerable reduction in the risk of coronary heart disease, myocardial infarction, and stroke. In addition, numerous randomized, controlled trials have confirmed that chocolate, especially dark chocolate containing large amounts of flavan-3-ols, improved risk factors for the constellation of conditions such as hypertension [6], [7], vascular endothelial dysfunction [8], [9], dyslipidemia [10], [11], and glucose intolerance [12], [13], that can contribute to metabolic syndrome. Several meta-analyses conducted after these clinical trials confirmed that dark chocolate could reduce the risk of cardiovascular disease [14]–[20]. In addition, recent cross-sectional studies have reported an inverse association between the frequency of chocolate ingestion and body mass index in healthy adolescents or adults [21], [22].

In our previous study [23], we confirmed that the respiratory exchange ratio (RER), where RER  =  carbon dioxide production (VCO_2_)/oxygen consumption (VO_2_), was significantly reduced as a result of the increase in lipolysis following repeated supplementation with flavan-3-ol fraction derived from cocoa (FL). In addition, repeated ingestion of FL increased concentrations of UCP a key protein of thermogenesis—in several tissues, and also augmented enzymes involved in *β*-oxidation such as carnitine palmitoyltransferase-2 (CPT-2) and medium-chain acyl-CoA dehydrogenase (MCAD). Moreover, we observed a significant increase in mitochondrial DNA copy number in skeletal muscle and brown adipose tissue (BAT) [23].

In this study, we compared the effects on energy expenditure of administering a single 10 mg/kg oral dose of FL with a 10 mg/kg dose of EC in mice, using indirect calorimetry and monitoring the initial biochemical changes responsible for thermogenesis and lipolysis in skeletal muscle and BAT. The changes in plasma catecholamine concentrations following treatment were also examined.

## Materials and Methods

### Materials

The flavan-3-ol fraction (FL) was provided by Meiji Co., Ltd (Tokyo, Japan) and was prepared from cocoa powder using a method described in a previous report [24]. In brief, the cocoa powder was defatted with n-hexane and the residue was extracted with acetone. The n-butanol-dissolved fraction of the extract was subsequently applied to a Diaion HP2MG column (Mitsubishi Kasei Co. Ltd., Tokyo, Japan). The fraction eluted with 80% ethanol was collected, freeze-dried, and used in the experiments. The concentration of catechins and procyanidins was determined by the method of high-performance liquid chromatography (HPLC). FL contained 4.56% (+)-catechin, 6.43% of (−)-epicatechin, 3.93% of procyanidin B2, 2.36% of procyanidin C1, and 1.45% cinnamtannin A2. (−)-Epicatechin was purchased from Tokyo Chemical Industry (Tokyo, Japan).

### Animals and diets

This study was approved by the Animal Care and Use Committee of the Shibaura Institute of Technology (Permit Number: 27-2956). All animals received humane care under the guidelines of this institution. Male ICR mice weighing 35–40 g were obtained from Charles River Laboratories Japan, Inc. (Tokyo, Japan). The mice were kept in a room with controlled lighting (12/12 hr light/dark cycles) at a regulated temperature between 23–25°C. A certified rodent diet was obtained from the Oriental Yeast Co., Ltd., Tokyo, Japan.

### Experimental procedures

During the first experiment of this study, the analysis of respiratory gas was performed during the feeding period. Four days after being fed a basal diet, the animals were divided into two groups; the animals in the control group (*n* = 13, Cont) were administered 4 ml/kg distilled water orally, whereas the mice in FL groups (*n* = 13) received 10 mg/kg FL via the oral route. Each animal was placed inside an open-circuit metabolic chamber for a 20 hr period, during which time they could eat ad libitum, at the same time their respiratory gas was being analyzed. In the second experiment in this study, the animals were treated with the vehicle (*n* = 8, Cont), FL (*n* = 8) or 10 mg/kg EC (*n* = 8) and their respiratory gas was analyzed over a 20 hr period of fasting. VO_2_ and excreted VCO_2_ were determined using a small animal metabolic measurement system (MK-5000RQ Muromachi Kikai Co. Ltd, Tokyo, Japan). The system monitored VO_2_ and VCO_2_ at 3-min intervals and calculated the RER using the RER  =  VCO_2_/VO_2_ formula. The VO_2_ and VCO_2_ measurements were converted to REE (kcal/20 hours, 8 hours of light cycle or 20 hours of dark cycle) using the Weir equation and the following formula: REE  =  (3.941 VO_2_ + 1.11 VCO_2_) * 1.44 * 60 min * hrs. To measure their spontaneous motor activity while they were sedentary, mice were placed one at a time in a chamber equipped with an infrared-ray passive sensor system (MMP10, Muromachi Kikai). This second experiment involved measurements being performed during the dark period (18:00 to 6:00) and light periods (12:00 to 18:00 and 6:00 to 8:00).

The third experiment required *n* = 8 animals per group to be euthanized under pentobarbital (50 mg/kg body weight IP) anesthesia (Tokyo Chemical Industry, Tokyo, Japan) 2, 4, and 20 hr after being administered their respective group's treatment in the absence of subsequent food. Tissues samples were collected by dissection and snap frozen in liquid nitrogen and stored at −80°C until analysis.

Plasma catecholamine levals were measured from blood collected with ethylenediaminetetraacetic acid (EDTA) during decapitation and exsanguination 1, 2, and 4 hr after treatments were administered as described above (*n* = 8 mice in each group). Plasma was stored at −80°C until analysis.

### Quantitative RT-PCR analysis

Total RNA was prepared from skeletal muscle and BAT using the TRIzol reagent (Life Technologies) according to manufacturer's instructions. In brief, 10 µg of total RNA was reverse-transcribed in a 20 µl reaction with high capacity cDNA Reverse Transcription kits (Applied Biosystems). Real-time reverse-transcription (RT)-PCR, using100 ng of total cDNA, was conducted using the StepOne Real-Time PCR System (Applied Biosystems). Primer and probe sequences were selected using a Taqman Gene Expression Assay (Applied Biosystems) and included the following gene and catalog numbers: GAPDH:Mm99999915_g1; UCP-1:Mm_01244861_m1; and UCP-3:Mm_00494077_m1; PGC-1α:Mm01208835_m1, all purchased from Applied Biosystems. Glyceraldehyde-3-phosphate dehydrogenase (GAPDH) was used as an internal control. The buffer used in the systems was THUNDER BIRD Prove qPCR Mix (TOYOBO). The PCR cycling conditions were 95°C for 1 min, followed by 40 cycles at 95°C for 15 s and 60°C for 1 min.

### Western blotting analysis

Tissues were homogenized in a microtube with lysis buffer (CelLytic MT cell lysis reagent; Sigma Aldrich, Japan) containing a protease inhibitor (Sigma Aldrich, Japan) and 0.2% SDS. Protein concentration was measured by the Bradford method. Protein (10 µg) was separated by SDS-PAGE using a 4–12% Bis-Tris gel and transferred onto a polyvinylidene difluoride membrane (Life Technology). The membrane was blocked with membrane-blocking reagent (GE Healthcare) for 1 hr. After blocking, the membrane was incubated with a rabbit polyclonal primary antibody against AMPK1α (1∶1600; sc-25792, Santa Cruz Biotechnology, Inc., USA), phosphorylated AMPK1α (1∶200; sc-33524, Santa Cruz Biotechnology, Inc., USA), antibody for 2 hr. After the primary antibody reaction, the membrane was incubated with appropriate horseradish peroxidase-conjugated secondary antibodies (1∶100000) for 1 hr. Immunoreactivity was detected by chemiluminescence using the ECL Select Western Blotting Reagent (GE Healthcare). Fluorescence band images were analyzed using Just TLC (SWEDAY) analysis software. Values of phosphorylated-AMPK1α were normalized to those for AMPK1α.

### HPLC analysis of plasma catecholamine concentrations

Plasma catecholamines were analyzed by HPLC-electrochemical detection (ECD) after being prepared with a monolithic silica disk-packed spin column (MonoSpin, GL Science, Tokyo Japan) [25]. Norepinephrine and epinephrine were obtained from Tokyo Kasei (Tokyo, Japan). Dopamine was acquired from Wako Pure Chemical (Topkyo, Japan). The 3,4-dihydroxybenzylamine (DHBA) used was from (Sigma Aldrich, Japan). Acetonitrile was purchased from Wako Pure Chemical (Tokyo, Japan). Plasma, 1 M phosphate buffer (pH 8.0) (50 µL), and 400 ng/mL DHBA (internal standard; 40 µL) were directly injected into the pre-activated spin column which was centrifuged at 3000 rpm for 5 min. The column was then rinsed with 200 µL of 100 mM phosphate buffer (pH 8.0) by centrifugation. Finally, the column was installed into a new microtube, and the analytes that were adsorbed onto the column were eluted with 1% acetic acid (200 µL). A 20 µL aliquot of the eluate was njected into the HPLC system (Prominance HPLC System Shimazu Corporation, Kyoto Japan) equipped with ECD (ECD 700 S, Eicom Corporation, Kyoto Japan) set at 650 mV. HPLC separation was conducted on an Inertsil ODS-4 (250×3.0 mm I.D., 5 µm) (GL Science) at 35°C, with a flow rate of 0.5 mL/min using a mobile phase comprised of 20 mM sodium acetate-citrate buffer/acetonitrile (100/16, v/v) containing 1 g/L sodium 1-octanesulfonate.

### Data analysis and statistical methods

All data were reported as the mean ± standard error. Statistical analyses were performed by Dunnett's post-test. A statistical probability of *P*<0.05 was considered significant.

## Results

### Resting energy expenditure and activity counts

**Figure 1:**
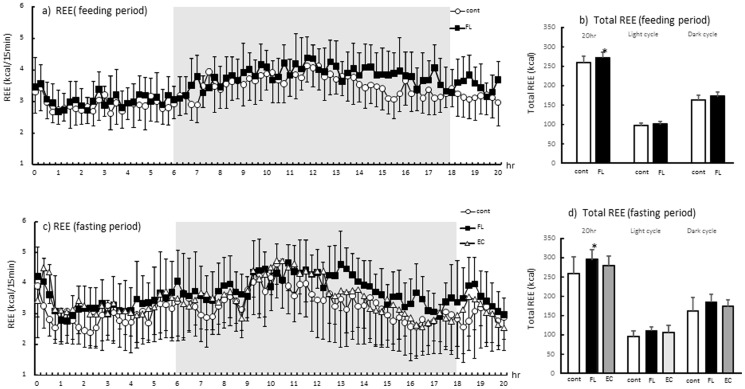
Respiratory energy expenditure (REE) 20 hr after administration of flavan-3-ols during ad libitum feeding (a) and the fasting period (c). Respiratory exchange ratio (RER) was calculated via oxygen consumption (VO_2_) and carbon dioxide excretion (VCO_2_) using the Weir equation. Total REE and light cycle (from 12:00 to 18:00 and from 6:00 to 8:00) or dark (from 18:00 to 6:00) REE was shown in (b; feeding period) and (d; fasting period). The animals were administrated vehicle (Cont, *n* = 13 during ad libitum feeding, *n* = 8 during fasting), 10 mg/kg FL (*n* = 13 during ad libitum feeding, *n* = 8 during fasting), or 10 mg/kg EC (*n* = 8 during fasting only). Values represent the mean ± standard deviation. Statistical analyses were performed by Dunnett's post-test. Significantly different from vehicle, *p<0.05.

The REE results are shown in Fig. 1. The data revealed over 20 hr after treatment of the chemicals (Fig. 1a, free feeding state; Fig. 1c, fasting state) and total and dark (from 18:00 to 6:00) or light (from 12:00 to 18:00 and from 6:00 to 8:00) cycle (Fig. 1b, free feeding state; Fig. 1d, fasting state). As shown in Fig. 1a and 1b, REE was marginally higher in FL compared with Cont throughout the measurement period during ad libitum feeding. There was a significant increase in REE during the total in FL compared with Cont in feeding period. In the fasting state, REE was high throughout the measurement period in the group treated with FL compared with those treated with vehicle, but this change or elevation in REE was not observed in the group treated with EC (Fig.1c). The REE was significantly elevated for the total following mixed FL treatment, but there were no such changes following EC treatment (Fig.1 d). There were also no significant changes in locomotor activity and RER among experimental groups (data not shown).

### UCPs and PGC-1α mRNA levels in BAT and the gastrocnemius

**Figure 2:**
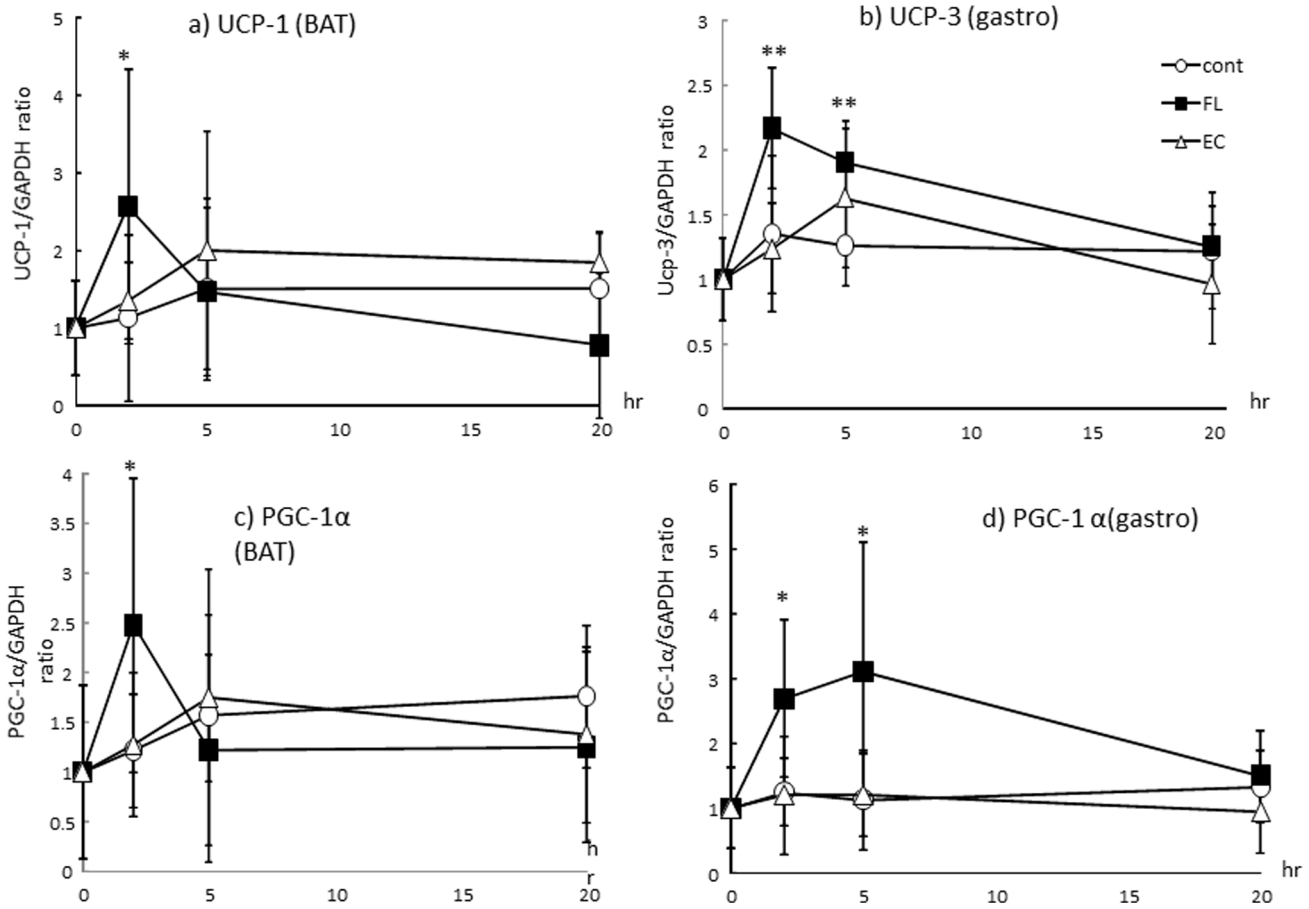
mRNA expression of UCPs and PGC1-αin BAT (a, b) or gastrocnemius (c, d) after administration of FL or EC. The animals were euthanized 2, 5, and 20 hr after administration of the vehicle (Cont, *n* = 8), 10 mg/kg FL (*n* = 8), or 10 mg/kg EC (*n* = 8). Values represent the mean ± standard deviation. Statistical analyses were performed by Dunnett's post-test. Significantly different from vehicle, *p<0.05, **p<0.01.

The change in mRNA expression of UCP-1 in BAT is shown in Fig. 2a. UCP-1 mRNA level was significantly increased 2 hr after administration of FL compared with that of Cont. mRNA expression of UCP-3 in the gastrocnemius was also significantly increased 2 and 5 hr after ingestion of FL (Fig. 2b). In contrast, there were no significant changes in UCPs following the administration of EC. PGC-1α mRNA levels in BAT and the gastrocnemius are shown in Fig. 1c and 1d. A significant increase in mRNA expression of PGC-1α was observed 2 hr after treatment with FL in BAT, and 2 and 5 hr after ingestion of FL in the gastrocnemius compared with mice in Cont. These changes were not observed in the mice treated with EC.

### Phosphorylation of AMPK1α in BAT and the gastrocnemius

**Figure 3:**
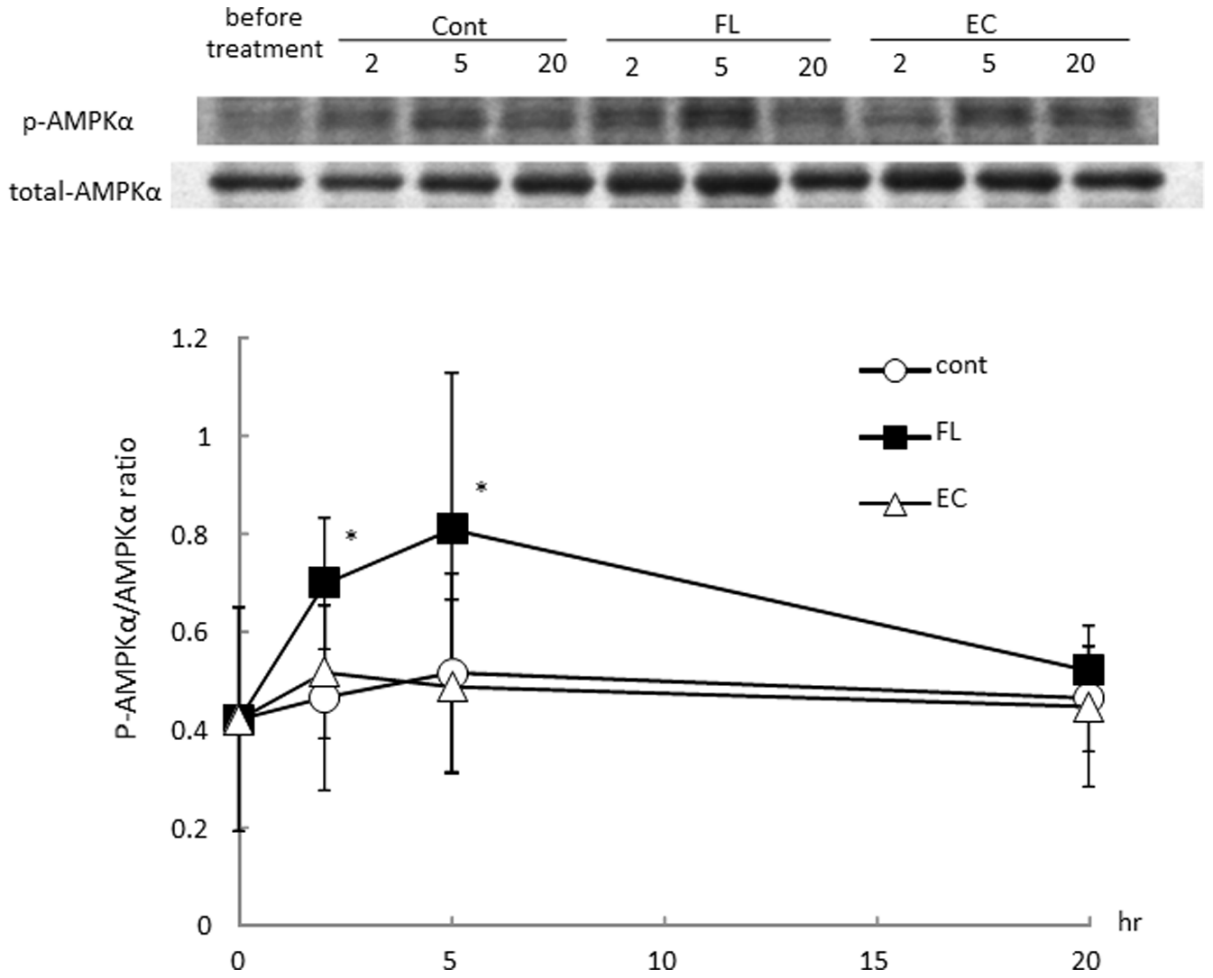
Phosphorylation of AMPK1αin BAT (a) or gastrocnemius (b) after administration of mixed flavan-3-ols or (−)-epicatechin. The animals was euthanized 2, 5, and 20 hr after administration of the vehicle (Cont, *n* = 8), 10 mg/kgFL (*n* = 8), or 10 mg/kg EC (*n* = 8). Values represent the mean ± standard deviation. Statistical analyses were performed by Dunnett's post-test. Significantly different from vehicle, *p<0.05, **p<0.01.

As shown in Fig. 3a, phosphorylated AMPK1*α* in BAT was increased 2 and 5 hr after treatment with FL. In the gastrocnemius, a significant elevation in phosphorylated AMPK1*α* occurred 2 and 5 hr after administration of FL (Fig. 3b). In contrast, no significant changes were evident in the EC-treated group of mice.

### Blood catecholamine concentrations

**Figure 4:**
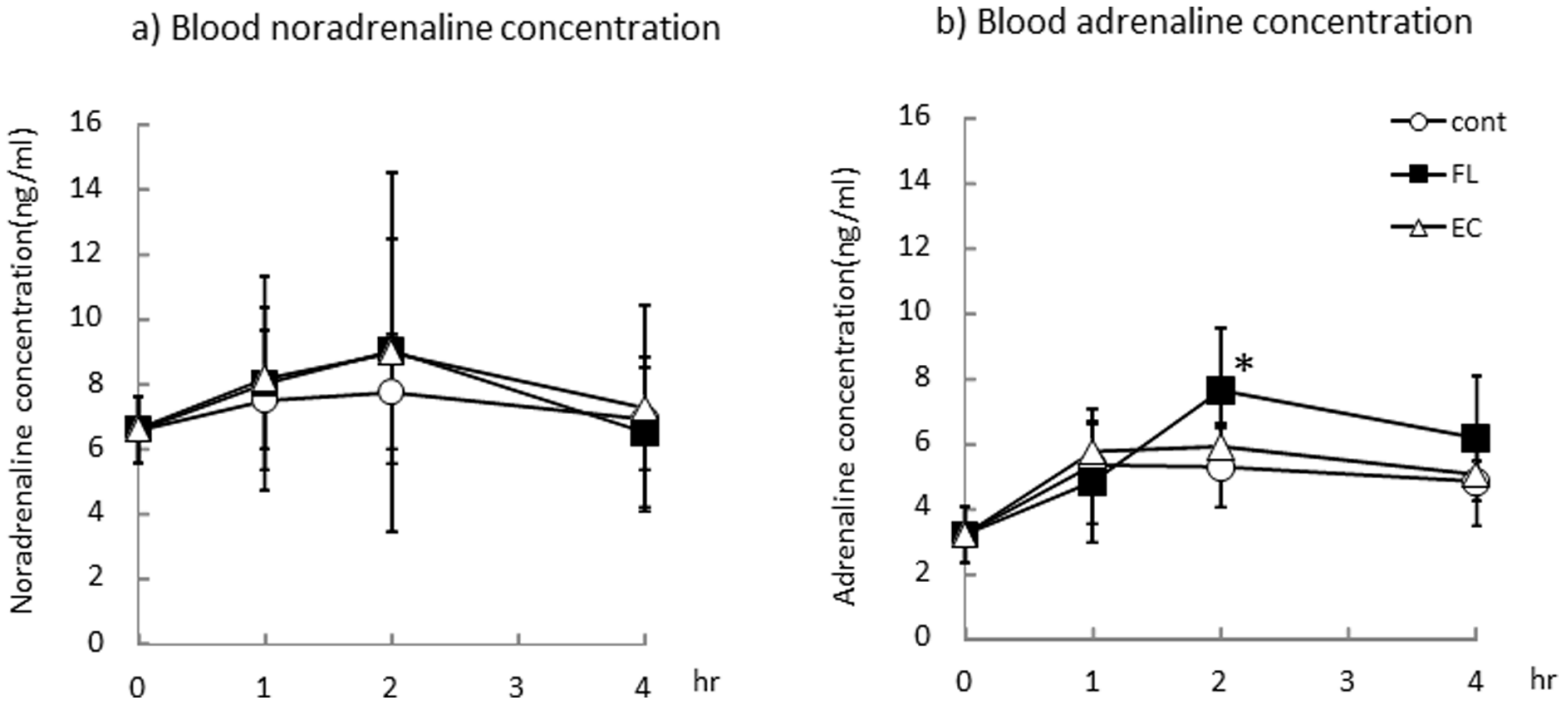
Blood noradrenaline (a) and adrenaline (b) concentrations after administration of mixed FL or EC. The animals were euthanized 2, 5, and 20 hr after administration of the vehicle (Cont, *n* = 8), 10 mg/kg of FL (*n* = 8) or 10 mg/kg of EC (*n* = 8). Values represent the mean ± standard deviation. Statistical analyses were performed by Dunnett's post-test. Significantly different from vehicle, *p<0.05.

The levels of the blood catecholamines adrenalin and noradrenalin 1, 2, and 4 hr after oral ingestion of the treatments by each group are shown in Fig. 4. There were no significant changes in plasma noradrenalin concentrations in any treatment groups during the experimental period. Plasma adrenalin concentrations in mice were significantly increased 2 hr after administration of FL versus Cont. No change in adrenalin concentrations were observed in the group of mice treated with EC.

## Discussion

Chocolate is known to be rich in flavan-3-ols. A previous report suggested that catechin and procyanidin contents (ranging from dimers to decamers) can be determined by HPLC [26], [27]. The flavan-3-ols fraction derived from cocoa (FL) used in this study contained 11% catechins and 7.7% procyanidins (ranging from dimers to tetramers). A single dose of a 10 mg/kg FL significantly enhanced REE both during ad libitum feeding and during a fasting period (Fig. 1). In contrast, a 10 mg/kg dose of EC did not result in any significant change in energy expenditure. A similar trend was observed in the mRNA expression of UCPs and PGC-1α in BAT and the gastrocnemius of mice. As shown in Fig. 2, a significant elevation in the mRNA expression of UCPs and PGC-1α was observed in the group of mice that received FL, but such a change was not evident in the mice treated with EC alone. Increases of REE were observed during 13 to 20 hours rather than primary measurement period, in contrast, mRNA expressions of UCPs or PGC1 α were observed 2 and 5 hours after FL treatment. It was suggested that REE increase by the treatment of FL may revealed protein synthesis induced by these mRNA changes. In addition, phosphorylation of AMPK1α was also increased following treatment with the FL, but not with the EC (Fig.3). According to these results, it was considered unlikely that EC was an indispensable component of FL, which as a mixture stimulated metabolic activity via the induction of UCPs and PGC-1α which are responsible for adaptive thermogenesis and lipolysis. It may also be indicated that these metabolic changes was synergic action of the catechins and procyanidins. Though 10mg/kg EC, which was more than 9 fold of catechins of 10 mg/kg FL, did not show any significant alteration. According to these results, it is possible that procyanidins rather than catechins contributed to these metabolic changes.

It is well-established that the bioavailability of catechins and procyanidins differ significantly. Catechins are present in the blood mainly as metabolites, such as conjugated forms with glucuronide and/or sulfate, and their absorption is reported to range between 10–20%. In contrast, unmetabolized catechins are nearly absent after ingestion [28], [29]. Procyanidins have been shown to be poorly absorbed via the gastrointestinal tract and are detected in very low concentrations in the blood [30], [31]. It was quite unlikely that procyanidins altered energy expenditure, based on their low bioavailability. In the present study, we found plasma adrenaline levels significantly increased after administration of a single dose of FL (Fig. 4). Adrenalin is secreted from the adrenal medulla and distributed in plasma, via the sympathetic nervous system, following stimulation in response to physiological or psychological stress [32]. Our data suggested the possibility that FL, especially procyanidins, similarly stimulate sympathetic nerves.

The sympathetic nervous system is known to play an essential role in the regulation of metabolic activity [33]. In BAT, the noradrenalin secreted from sympathetic nerve terminals binds to a β3 adrenergic receptor and induces UCP-1, which is responsible for thermogenesis [34]. It has been suggested in reports of exercise and in agonist studies, that in skeletal muscle, stimulation of the β2 adrenalin receptor upregulates PGC-1α [35], [36] and can induce the UCP-3 activity involved in adrenergic effects [37], [38]. In our previous reported study, mean blood pressure and heart rate were transiently increased soon after treatment with a single dose of flavan-3-ols, and also increased blood flow in the cremaster muscle [39]. The autonomic nerves are also known to play a crucial role in the circulatory system. Stimulation of sympathetic nerves induced a transient increase in blood pressure through the α1 adrenergic receptors in vascular smooth muscle, and heart rate was affected through β1 adrenergic receptors in cardiac muscle. These results suggested that after a single dose of FL, not only can metabolic changes occur, but also hemodynamic alterations induced by sympathetic nerve stimulation are possible.

Yamashita et al. reported a significant increase in phosphorylated AMPK1α after treatment with flavan-3-ols [40], and we have confirmed a change of this nature in the present study (Fig.3). Previously, our research revealed an increase in cremasteric-recruited capillary number, which indicated a requirement for O_2_ during ATP production, as a result of a single dose of flavan-3-ols [39]. Hypoxia-induced phosphorylation of AMPK1α in skeletal muscle has been reported previously [41], and while it is possible that hypoxia occurring after FL treatment in skeletal muscle can induce such a change, further experiments are needed to determine the mechanism for enhancement of AMPK1α phosphorylation.

We found a significant increase in mitochondrial number and *β*-oxidation in the gastrocnemius, soleus, and BAT and decrease in RERs after 2 weeks of flavan-3-ols feeding [23]. PGC-1α is recognized as a master regulator of mitochondrial biogenesis [42]–[44] by activating respiratory chain and fatty acid oxidation genes, increasing mitochondrial number, and enhancing mitochondrial respiratory capacity. It was suggested that the initial changes responsible for metabolic activity, such as that of PGC-1α, contributed to the alterations in this murine phenotype.

In conclusion, we found that FL significantly enhanced systemic energy expenditure, accompanied with an increase in gene expression associated with thermogenesis and lipolysis; however, EC exhibited this less robustly or effectively. In addition, it was suggested the possible interaction between metabolic changes and the increase in plasma catecholamine concentrations after administration of a single oral dose of FL. These effects may be able to mitigate the risk of metabolic syndrome.
